# Assessing the Psychometric Properties of the Internet Addiction Test in Peruvian University Students

**DOI:** 10.3390/ijerph17165782

**Published:** 2020-08-10

**Authors:** Arnold Alejandro Tafur-Mendoza, Julio César Acosta-Prado, Rodrigo Arturo Zárate-Torres, Duván Emilio Ramírez-Ospina

**Affiliations:** 1 Research Center (CIUP), Universidad del Pacífico, Lima 15072, Peru; aa.tafurm@up.edu.pe; 2 School of Business Science, Universidad del Pacífico, Lima 15072, Peru; 3 School of Accounting, Economic and Business Sciences, Universidad de Manizales, Manizales 170001, Colombia; merca2@umanizales.edu.co; 4 Colegio de Estudios Superiores de Administración, Bogota 111071, Colombia; rodrigo.zarate@cesa.edu.co

**Keywords:** internet addiction test, university students, Peruvian sample, psychometric properties

## Abstract

The use of the Internet has been gradually and unstoppably gaining ground in all areas of life, from recreational activities to how social relations are established. However, the existence of clinical cases indicates that the addictive use of the Internet is a problem that seriously affects some people. Among the instruments that measure this construct, the Internet Addiction Test (IAT) stands out. However, instrumental studies of this test are scarce in Latin America. The present study sought to analyze the psychometric properties of the IAT in a sample of 227 Peruvian undergraduate university students. Confirmatory factor analysis was used to provide validity evidence based on the internal structure, and evidence based on the relationship with other variables was also provided. Reliability was estimated through the ordinal alpha coefficient. The results indicated that the IAT adequately fits a bifactor model (with two specific factors, time/control and stress/compensate), obtaining good levels of reliability. Additionally, the IAT scores correlate significantly with the average number of hours per day on the internet and social skills. The results lead to the conclusion that the scores in the IAT have evidence of validity and reliability for its use.

## 1. Introduction

In the framework of a society in which both communication and the free flow of information are closely related to the development of the network, it is necessary to know to what extent reality and the virtual sphere intermingle. From the lowering of costs, the multiplicity of ways of accessing the network, and the advent of social networks, the Internet grows exponentially every day [1]. As studies in Sweden [2] and Spain [3] seem to indicate, the fierce proliferation of the network as a means of communication has brought negative consequences (cyberbullying, problematic Internet use, sexting, nomophobia, etc.) that have a stronger impact on the young population, having identified a series of problems among which stand out the addiction to this environment and that affect above all the social sphere of the individual.

However, the use of the Internet has expanded gradually and unstoppably in all areas of life [4]. The existence of clinical cases indicates that maladaptive use of the Internet is an existing problem that seriously affects some people, mainly those with special emotional needs and young people and adolescents [5]. The use or abuse of the Internet arises from disciplines, such as psychology or psychiatry [6]. In this sense, it is not surprising that the term Internet addiction was used for the first time by the psychiatrist Ivan Golberg in 1995 [7]. The literature on problematic use of the Internet shows a very varied terminology to describe the problems derived from the use of the Internet, among which are: Computer addiction, excessive use of the Internet, pathological use of the Internet, Internet dependence, compulsive use of the Internet, disorder by impulsive use, and compulsive use of the Internet or Internet addiction [8,9].

Kimberly Young [10], after reviewing a series of investigations, which indicated that some online users became addicted to the Internet in the same way that others became addicted to drugs, alcohol, or gambling, suggests the need to empirically investigate the concept of addictive use of the Internet. In addition to the review, the study sought to identify whether excessive use of the Internet can be considered addictive and to know the magnitude of the problems created by these abuses. However, Young’s approaches to Internet addiction sparked a controversial debate among doctors and academics at the time [11].

On the other hand, there are proposals [12] that suggested that Internet addiction is a disorder that should have been considered in the fifth edition of the Diagnostic and Statistical Manual of Mental Disorders (DSM-V) since it is an impulsive-compulsive spectrum disorder [13], which consists of the use of computers. However, these recommendations or proposals were not reflected in the DSM-V, since the term Internet addiction does not appear in the manual [14]. Among the diagnoses referenced in the DSM-V, Internet gaming disorder (IGD) is strongly related to the pathological nature of Internet use. The excessive use of the Internet not only involves the reproduction of online games (for example, it also implies the excessive use of social networks, such as Facebook or Twitter). Therefore, IGD is within the Internet addiction. Thus, research on other excessive uses of the Internet should follow guidelines analogous to those suggested with IGD [14].

Among the theoretical models proposed to understand Internet addiction, Davis’ cognitive-behavioral model [15] is the one that has had the most development and has been empirically tested in different contexts [16]. Two parts are distinguished in this model, one specific and the other general. The first involves excessive use of an aspect of the internet, while generalized use encompasses multiple excessive uses of the Internet [15]. Generalized use is associated with social support and social service uses of the Internet, where maladaptive cognitions are a strong predictor of this component and to a lesser extent of specific use [17]. Under this model, symptoms of Internet addiction are primarily affective or behavioral and are usually preceded or caused by cognitive symptoms, mediated by specific and general pathological Internet use [15,16]. The value of the cognitive-behavioral model lies in that it contemplates a continuum of severity regarding Internet use, allowing a better understanding by mental health specialists of the way and degree to which the excessive use of the internet can affect the lives of people [18].

To measure Internet addiction, various tests have been developed, which, mainly, are based on the diagnosis of this disorder, including the Problematic Internet Use Questionnaire (PIUQ) [19], the Compulsive Internet Use Scale (CIUS) [20], Internet-Related Problem Scale [21], and the Internet-Related Experiences Questionnaire (IREQ) [22]. The PIUQ, made up of 18 items, was built for the general population and measures problems related to Internet use through three subscales: Obsession, neglect, and control disorder. The CUIS is made up of 14 items that measure the severity of compulsive Internet use. The Internet-Related Problem Scale has 20 items that address DSM-IV symptoms for substance abuse: Tolerance, escape from other problems, reduced activities, loss of control, negative effects, withdrawal, craving, and introversion. The IREQ was developed in middle-school students and is made up of 10 items that measure possible Internet addiction based on intrapersonal and interpersonal experiences. However, the test that has had the most development and that has been adapted to various contexts is the Internet Addiction Test (IAT) [23]. Thus, the objective of this study was to analyze the psychometric properties of the IAT in a sample of Peruvian undergraduate university students.

## 2. Literature Review

For this study, in a search conducted in Scopus and Web of Science (WoS) on studies of the psychometric properties of the IAT, 46 studies were found (April 2020). Table 1 presents a summary of 39 of the 46 studies found, which were those that analyzed the internal structure of the IAT, an aspect that does not have a consensus in all the studies carried out. Most of the studies were carried out in Europe and Asia; in North America, only one study was found in the United States and another in Canada, while, in Latin America, the IAT has been studied in Colombia and Chile. Regarding the populations where these studies have been carried out, the majority were in university students, probably due to the accessibility of the sample and that it is the age group that spends the most time on the Internet (young people between 18 and 25 years old).

The reliability of the IAT has usually been estimated through the alpha coefficient, obtaining, in most cases, acceptable values above 0.70, and even exceeding 0.90. However, most of these studies have not considered the assumptions that this coefficient has, such as the one-dimensionality of the data, that the measurement model is tau-equivalent, and that there are no correlated errors [63]. Violation of any of these assumptions would distort the values reported in these studies. Additionally, there is no consideration of the ordinality of the items, which must be considered when working with Likert-type scales [64]. A meta-analysis of the internal consistency of the IAT (reliability generalization) indicates a combined alpha of 0.90 for samples of university students [65].

Regarding the internal structure of the IAT, the literature shows factorial models with one to six factors. Although, the diversity of models increases, because two studies may have the same number of factors, but they differ in the number of items used (not all studies end their analyses with the original 20 items but eliminate some of them because they do not fit their proposed model), in the addition of correlated errors (in many cases to improve the adjustment indices in the confirmatory factor analysis, without taking into account the substantive justification of the correlated errors [66]) or in the distribution of the items by the factor (two models can coincide in the number of factors and items, but the grouping of the items is different). This large number of models does not allow for a uniform IAT structure to be available, and for studies seeking to study this aspect in a particular sample, they have to resort to exploratory techniques or to test a large number of existing models.

An aspect linked to the above is the analysis technique used to find the factor structure in these studies. Almost half of the studies reported in Table 1 indicate that the principal component analysis (PCA) has been performed as the first or only technique, which is not suitable for psychological constructs, since it is a variable reduction technique; therefore, it seeks to explain the greater proportion of explained variance, considering both the variance shared by the items and the error variance. On the contrary, the factor analysis only considers the extraction of the factors the variance that the items share among themselves [67]. The use of the PCA could lead to obtaining structures that do not fit the data, especially since the measurement nature of the data is not considered.

In Peru, only one study of the psychometric properties of IAT [68] in middle-school students between the ages of 13 and 19 years was found. Item 1 was removed from the scale because it presented a low discriminatory capacity; therefore, this version had 19 items. Reliability was estimated through the alpha coefficient, obtaining a value of 0.870. On the other hand, the internal structure was evaluated by PCA, finding a factorial solution of four components. This structure was corroborated in the same sample through confirmatory factor analysis (CFA). It is important to note that the use of middle-school students and university students is different. University students use the Internet primarily for information search; also, they spend more time online and have a wider range of Internet uses than middle-school students [69].

Another source of validity evidence that has been explored in these background studies is evidence based on the relationship with other variables, specifically convergent evidence, where the hours of daily Internet use was used as a measure, in addition to the IAT [38,41,45,50]. In these studies, correlation coefficients between 0.29 and 0.48 were found. Additionally, Internet use has an impact on a person’s social life, and studies have shown that Internet addiction is negatively related to social skills [70,71,72].

Considering all the above, the IAT is one of the most used instruments for measuring Internet addiction and that, in Peru, it has only worked with middle-school students in the adolescence stage but not in other samples, such as university students, which is precisely in this group where most adaptations have been made in other countries. Furthermore, the statistical procedures used in previous studies may be questionable and, in some cases, inadequate. Thus, the IAT does not have a version for Peruvian university students, so its psychometric properties are not known in this population and the extrapolation of results in other contexts would not be appropriate, since validity and reliability are not inherent in a test but correspond to the specific interpretations and uses of the scores obtained in a test [73]. Therefore, the objective of this study was to analyze the psychometric properties of the IAT in a sample of Peruvian undergraduate university students.

## 3. Materials and Methods

### 3.1. Design

According to the classification system of research designs in psychology [74], the objective of the study corresponds to an instrumental investigation, since the psychometric properties of a psychological instrument are analyzed in a specific sample. For the development of the study, various standards, guidelines, good practices, and recommendations were followed in instrumental studies in the behavioral and health sciences [73,75].

### 3.2. Participants

**Figure 1:**
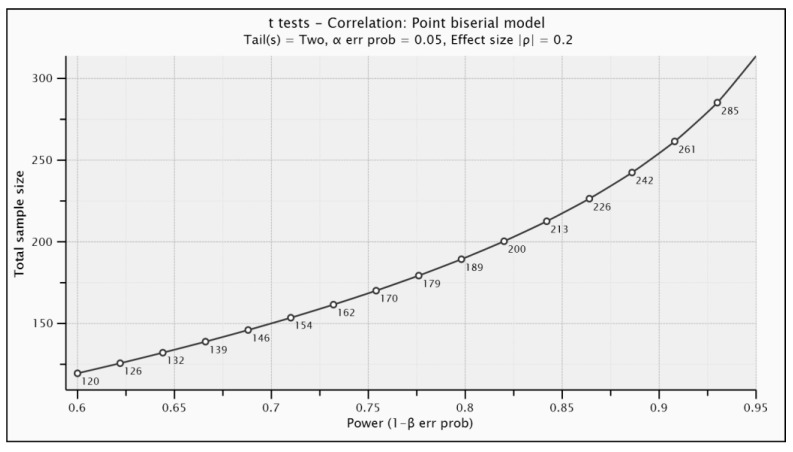
A priori statistical power analysis to determine the minimum recommended sample size.

The selection of the participants was carried out through an intentional non-probability sampling [76], where the individuals in the sample were directly and selectively chosen, seeking to obtain similar proportions in each of the categories of the variables sex, year of study, and academic discipline. Regarding the sample size, a priori statistical power analysis was performed to determine the minimum recommended sample size. Statistical power is the ability of a statistical test (for example, U Mann–Whitney or H Kruskal–Wallis) to reject a null hypothesis when it is false; in other words, it is the probability of not committing the type II error [77]. The input parameters for this analysis were based on a simple two-tailed correlational model (similar to the one that will be carried out in the collection of validity evidence based on the relationship with other variables), the significance level (α) being 0.05, the expected effect size equal to 0.20 (recommended minimum value representing practical significance in social science data [78]), and an expected statistical power of 0.80 (recommended minimum in behavioral science [79]). The recommended minimum sample size was 191 (Figure 1).

The final sample was made up of 227 Peruvian undergraduate university students from a public university located in Metropolitan Lima (Peru). The ages of the participants were between 18 and 40 years (M = 20.81, SD = 2.92). The highest proportion of students were females (57.30%), in their second year of studies (30.00%), and belonged to professional careers in the engineering area (19.80%). Table 2 presents a more detailed description of the sample characteristics.

### 3.3. Instruments

#### 3.3.1. IAT—Internet Addiction Test

The Spanish version of the IAT was used (Appendix A), adapted to a sample of Colombian university students [32]. The IAT is made up of 20 items, all in a positive sense, on a five-point Likert-type scale (1 = rarely; 2 = occasionally; 3 = frequently; 4 = usually; 5 = always), the minimum score being 20 and the maximum score 100, where the student was asked to choose the alternative that best suits their reality. The IAT items were reviewed by the authors of this study, pointing out that it was not necessary to adjust their wording. To corroborate this assumption, a pilot study was carried out with 10 university students, who indicated that they had no problems in understanding the items.

In the adaptation study, the total scale had a good level of reliability (α = 0.89). Likewise, through an analysis of the main components, they found an internal structure of three factors (consequences for the use of the Internet, cognitive-emotional dimension, and time control), which together had an explained variance of 47.80%, and correlated positively with the number of hours of daily Internet access, although the magnitudes were low [32].

For the application of this study, in addition to the IAT, a sociodemographic and Internet usage file was added to collect information on the career that the participants studied, as well as the year of studies they were studying, age, gender, and the average daily hours spent on the Internet.

#### 3.3.2. EHS—Social Skills Scale

The Social Skills Scale, developed by Gismero [80], is made up of 33 items. Five items are written in the direct sense, while 28 items are in an inverse sense, seeking to detect the lack of assertion or deficit in social skills. The original instrument is composed of six factors: (1) Self-expression in social situations, eight items; (2) defense of one’s rights as a consumer, five items; (3) expression of anger or disagreement, four items; (4) assertiveness, six items; (5) making requests, five items; and (6) starting interactions with the opposite sex, five items. Each item is answered using a four-point response format (A = I do not identify myself at all; B = Rather it does not have to do with me, even if it ever happens to me; C = It describes me approximately, although I do not always act or feel like this; and D = Strongly agree, and I would feel or act like this in most cases). A higher global score indicates that the person has a higher level of social skills and better insertion in various contexts or situations.

This instrument has been studied in various countries, presenting adequate psychometric properties, good levels of reliability, adequate adjustment to the six-factor structure, and discriminant evidence [81]. In the present study, the scores on the items showed good internal consistency at the global level (ω = 0.890). Likewise, in the factors, the omega coefficient (ω) varied from 0.500 to 0.748.

### 3.4. Procedure

The data collection process began with the request of permits, both to the directors of the schools of each academic discipline and the teachers in charge of some courses for the application of the instrument in classrooms. Before the application of the IAT, the students were given an informed consent form that contained the objective of the evaluation and where they were guaranteed the confidentiality and anonymity of their answers, as well as the possibility of withdrawing from their participation at any time of the evaluation without consequence. Only those students who voluntarily signed the informed consent participated in the study.

The application was collective, with an average duration of 15 min. Data collection lasted approximately five weeks. At the end of each evaluation, the examiners reviewed the application protocols to verify that there are no unanswered items or items with more than one marked answer option. If one or both situations described occurred, the evaluated person was asked to correct and provide their definitive answer. After data collection, each evaluation protocol was coded for the elaboration of the database in a Microsoft Excel 2016 spreadsheet.

During the evaluation process, the ethical guidelines for working with humans outlined in the code of ethics of the American Psychological Association (APA) were followed. Additionally, the ethical principles of the Declaration of Helsinki were respected, including its recent updates and regulations for research on human beings.

### 3.5. Data Analysis

The statistical analysis was divided into four stages: (1) Item analysis; (2) validity evidence based on the internal structure of the test through CFA; (3) validity evidence based on the relationship with other variables from the convergent evidence; and (4) reliability analysis using the internal consistency method.

The descriptive analysis of the items was performed using the mean and standard deviation, as measures of central tendency and dispersion, respectively. As descriptive measures of the distribution of the items, the skewness and kurtosis coefficients were used, where values between −2.00 and 2.00 indicate that the items follow a normal distribution [82]. Likewise, item discrimination was estimated through the item-rest polyserial correlation, which considers the ordinality of the items, considering values above 0.30 as adequate [83]. Finally, possible floor and ceiling effects in the items were analyzed, that is, to identify the proportion of participants who chose the lowest alternative (floor effect) and the highest (ceiling effect), taking as acceptable effects those that were between 1% and 15% [84].

The CFA was carried out using the 23 models presented in Table 1, considering only those models made up of the 20 original items and whose factors had at least three items. The estimation method used was the weighted least squares with mean and variance adjusted (WLSMV), appropriate for observable ordinal variables and that performs well against slightly non-normal underlying distributions [85]. The WLSMV involves the use of the diagonal weighted least squares (DWLS) estimator with robust standard errors and a statistical test with adjusted mean and variance (using a scale-shifted approach). To assess the level of adequacy of the models, the following fit indices were used [86,87]: SSχ^2^/*df* < 2.00, root mean square error of approximation (RMSEA) < 0.08, comparative fit index (CFI) > 0.90, Tucker–Lewis index (TLI) > 0.90, standardized root mean square residual (SRMR) < 0.08, and weighted root mean square residual (WRMR) < 1.00.

The convergent evidence involved correlating the scores obtained in the IAT with another measure that seeks to evaluate the same construct or other construct with which it is expected is correlated; in this case, these measures were the average of daily hours that the study participants spent on the Internet and a social skills scale. Before selecting the correlation statistic to be used, the presence of outliers in these two variables with which the IAT scores were correlated was examined. Univariate analysis of outliers was visually inspected through boxplots for each variable (including the six dimensions of the social skills scale). A few outliers were found in two factors of the social skills scale and in the average of daily hours that the study participants spent on the Internet. Therefore, a robust statistic was chosen for the correlation between variables, the skipped correlation coefficient. This coefficient is robust to slight changes in any distribution and has the advantage of treating outliers in a way that considers the general structure of a data set [88]. As a measure of the effect size, the coefficient of determination was used, considering its interpretation values of 0.04, 0.25, and 0.64 as the minimum, moderate, and strong effect, respectively [78].

The internal consistency of the items was estimated using the ordinal alpha coefficient [89] since it works with the inter-item polychoric correlation matrix. Values above 0.70 were considered acceptable [90].

A priori statistical power analysis to determine the minimum sample size required for the study was performed using the G*Power 3.1.9.7 software [91]. The other analyses were performed in the R software version 4.0.2 (R Foundation for Statistical Computing, Vienna, Austria, 2016) [92], using the following packages: pacman 0.5.1 [93], readxl 1.3.1 [94], tidyverse 1.3.0 [95], psych 1.9.12.31 [96], lavaan 0.6–6 [97], BifactorIndicesCalculator 0.2.0 [98], semTools 0.5–2.925 [99], and WRS 0.36 [100].

## 4. Results

### 4.1. Item Analysis

Table 3 presents the results of the item analysis. The means of the items were between 1.352 (item 20) and 3.084 (items 1 and 7), indicating that the participants’ responses were concentrated on the lowest alternatives (rarely, occasionally, and frequently). Likewise, the standard deviation fluctuated between 0.658 (item 20) and 1.211 (item 7), showing low variability in the data. Regarding the skewness and kurtosis measures, most of the items presented values between −2.00 and 2.00. However, items 9, 15, 19, and 20 showed an excess of kurtosis, and these last two also indicated an excess of skewness. Therefore, these four items showed a deviation from a normal distribution.

Concerning the item-rest polyserial correlation, all the items were above the threshold of 0.30, ranging from 0.371 (item 7) to 0.684 (item 15), indicating good discrimination by the IAT items. On the other hand, in the analysis of the response options, a soil effect was found in all the items, except for items 1 and 7. However, regarding the ceiling effect, most of the items had acceptable values (between 1% and 15%). These effects reflect the tendency of the participants to choose the lowest response options, being the most prominent in items 19 and 20, where more than 70% of the sample chose the response option “rarely”. On the contrary, the “always” alternative was selected in a low percentage, even reaching 0% in some items (items 6, 8, 10, 11, 15, and 20).

### 4.2. Validity Evidence Based on the Internal Structure

The models tested were taken from Table 1. However, of the 39 studies presented, the studies selected were those where the factor structure was configured by all the items of the IAT (20 items) and that the proposed factors have at least three items in its composition, which is recommended to achieve an adequate representation of a factor [86]. Twenty-four studies met the two requirements and their factor models were tested. Four studies had the same unifactorial structure [29,45,51,53]. Whereas, five models [26,32,38,46,59] presented problems in the analysis: Covariance matrix of latent variables was not positive definite [59], some estimated latent variable variances were negative [26,38,46], or the model did not converge [32].

**Figure 2:**
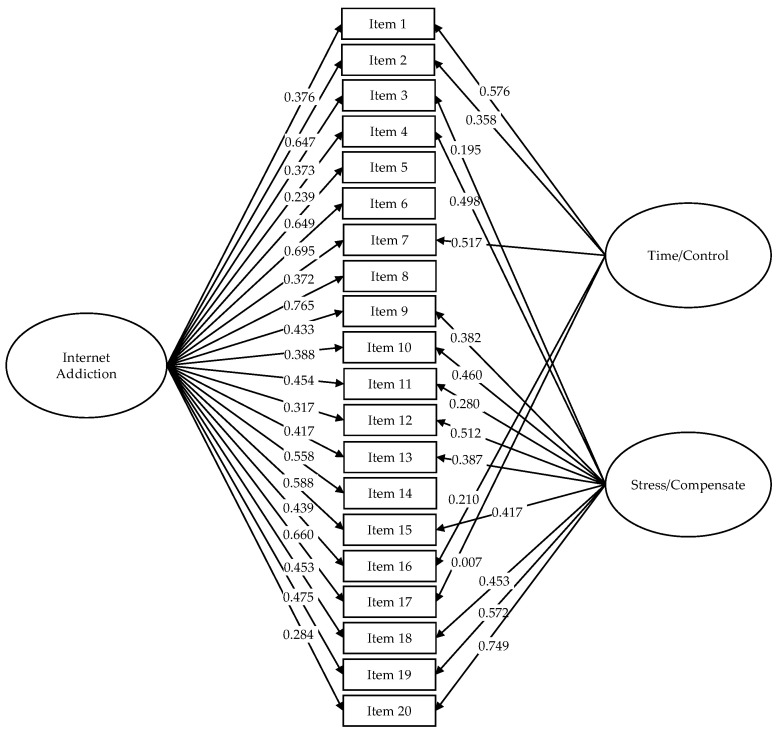
Factorial structure of the bifactor model.

Table 4 presents the fit indices of the models tested. The bifactor model of Watters et al. (2013) [34] was the one that obtained the best results (SSχ^2^/*df* < 2.00, RMSEA < 0.08, CFI > 0.90, TLI > 0.90, SRMR < 0.08, and WRMR < 1.00). Figure 2 shows the factor structure of the bifactor model, made up of a general factor that measures Internet addiction through 20 items and two specific factors, time/control and stress/compensate, the first factor consisting of five items (1, 2, 7, 16, and 17) and the second factor of 11 items (3, 4, 9, 10, 11, 12, 13, 15, 18, 19, and 20). Figure 2 also presents the factor loadings of the items in the general factor and the corresponding specific factors.

Additionally, complementary statistical indices were calculated that allowed assessment of the robustness of the general factor, as well as the contribution of specific factors [101]. The omega hierarchical was used for the general factor and the specific factors, expecting values above 0.70 (ω_H_) for the first and greater than 0.30 (ω_HS_) for the seconds. The results indicate a value of 0.704 for the general factor, 0.234 for time/control, and 0.478 for stress/compensate. The H coefficient was also calculated, considering values greater than 0.70 to be adequate. The general factor obtained a coefficient of 0.888: For the time/control factor it was 0.785 while, for the stress/compensate factor, the H coefficient was equal to 0.513.

The explained common variance (ECV) for the general factor was 0.612, while the factors (ECV of a specific factor concerning itself) showed an ECV of 0.368 for time/control and 0.562 for stress/compensate. On the other hand, the proportion of uncontaminated correlations (PUCs) was equal to 0.658. At the item level, the ECV index (I-ECV) was between 0.126 and 1.000. Thus, the results allow the use of this bifactor model [102].

### 4.3. Validity Evidence Based on Relations to Other Variables

The convergent evidence was collected from the correlation between the two specific factors of IAT and the general factor with the average hours per day that study participants spent on the Internet and social skills. The minimum time of hours per day was less than one hour and the maximum 12 h (M = 3.368, SD = 2.277). The skipped correlation coefficients between the average daily hours on the Internet with Internet addiction (and time/control factor) were statistically significant (Table 5). Likewise, the effect size (skipped correlation coefficient squared) in the time/control factor and total Internet addiction was greater than 0.04, indicating a recommended minimum effect size that represents practical significance in social science data [78]. Regarding social skills, the correlations between Internet addiction (and its two factors) with social skills (and the defense of rights factor) were statistically significant (Table 5). Similar results were obtained for correlations between Internet addiction (and stress/compensate factor) with self-expression, disagreement, and assertiveness (Table 5). The size of the effect between Internet addiction and social skills presented practical significance. These findings provide convergent evidence to the IAT.

On the other hand, the correlation between the specific factors of the IAT presented a minimum effect size (skipped correlation coefficient squared > 0.04). For the correlation between time/control and stress/compensate with Internet addiction, the effect size was strong (skipped correlation coefficient squared > 0.64). In all three cases, the correlations were statistically significant (Table 5).

### 4.4. Reliability

Reliability was evaluated by the internal consistency method, using for this purpose the ordinal alpha coefficient, which considers the ordinal nature of the items for its calculation. The specific factors and the general factor obtained satisfactory values, above 0.70 (Table 5). Likewise, the coefficients obtained were not remarkably high (0.90 or higher), indicating that the IAT, in the sample studied, does not include redundant items [103].

## 5. Discussion

The present study analyzed the psychometric properties of the IAT in a sample of Peruvian university students. The 20 items that make up the instrument presented adequate levels of discrimination, although the analysis of the response options indicated the presence of a floor effect in 18 items. Likewise, the levels of skewness and kurtosis were acceptable in most of the items. Regarding the validity evidence based on the internal structure, different models were tested through CFA, being the bifactor model with two specific factors (time/control and stress/compensate), the one that presented the best indexes of adjustment. Another source of validity evidence that was used was that based on the relationship with other variables, specifically the convergent evidence, finding statistically significant correlations between the two specific factors and the general factor with the average number of hours per day on the Internet and social skills. Finally, the reliability, estimated through the ordinal alpha coefficient, was acceptable for the general factor and the specific factors.

The items showed discrimination indexes above 0.30, which implies that each item is related to the other items taken together, which would also justify the presence of an underlying general factor. These results agree with a previous study [43], where all the items were higher than the cut-off point used in this study. However, other studies showed problems only with item 7 (“How often do you check your email before something else that you need to do?”), finding values of 0.170 [42], 0.250 [41], −0.098 [104], and 0.195 [32], while all the other items were greater than 0.30. In this study, item 7 had the lowest item-rest correlation (0.371).

Item 7 is problematic in the literature because it would be mainly relevant for university students or people whose jobs involve communication by this means. In university students, virtual communication occurs mainly with their professors by email, for sending papers, receiving corrected papers, or notifying activities on virtual platforms, as well as with their peers to exchange information (e.g., articles or books) or share working documents. It is important to highlight that, in this study, the item-rest correlation was estimated using the polyserial correlation, unlike previous studies that worked with the Pearson correlation coefficient. The polyserial correlation coefficient considers the ordinality of the items, being more precise in estimating the degree of item discrimination.

The presence of a floor effect in the items, together with low averages in these, shows the low level of Internet addiction of the participants. This result may be due to the characteristics of the study participants, who belong entirely to a public sector university, where most of the population belongs to a medium or medium-low socioeconomic level, having some limitations regarding the Internet accessibility as it involves spending on devices (cell phones, tablets, laptops, etc.) and mobile data for connectivity. On the other hand, Internet addiction is a clinical construct, so its presence in a non-clinical population (university students) should be low under normal conditions.

Regarding the internal structure of the IAT, this study recollected a large part of the models reported in previous studies to test them and find out how the IAT is structured in the Peruvian sample. The bifactor model (one general factor and two specific factors) [34] presented the best fit. In the reviewed literature, the other study that reported a bifactor model found a general factor and three specific factors [56]. Comparing the results obtained in this study with those reported by Watters et al. [34], many points of agreement were observed, both at the level of fit indices and in factor loadings. In both studies, the factor loadings had higher values in the general factor than in the specific factors. In both studies, several items presented factor loadings below 0.30 or 0.40, which are the usual cut-off points in this type of study. However, the results of the present bifactor model indicate that those items that had low factor loadings in the general factor, had higher factor loadings in the specific factors, and vice versa.

The characteristic described above is typical of bifactor models, which allow the simultaneous evaluation of the influence of the general factor and specific factors on the variability of each item. The evaluation of the bifactor model through the omega hierarchical (ω_H_ and ω_HS_) and H coefficients (also known as construct reliability), ECV, I-ECV, and PUC, provided evidence regarding the relevance of the model. The remaining 15 models reviewed and tested presented convergence or adjustment problems. The difference in fit between the previous studies and the present study is probably due to the different estimation methods used. In this study, the WLSMV estimator was used that considers the categorical nature of the items. Furthermore, many of the previous studies had problems in choosing the appropriate statistical technique or methods.

The use of the PCA for work with psychological variables is not appropriate since in its conception it considers formative models, useful in other disciplines (economics, marketing, among others), where it seeks to group indicators or reduce the number of variables. By contrast, factor analysis works with reflective models, where an underlying variable (factor) causes certain behaviors (indicators or items). For the factor analysis, the exploratory or non-restrictive version involves making a series of decisions during the analysis, the most critical being the determination of the number of factors. Additionally, the choice of the rotation method should be justified by how the factors are related. In the reviewed antecedents, the use, in most of the studies, of the “Little Jiffy” was observed, which supposes a routine of analysis in the three mentioned aspects: PCA, eigenvalues greater than one (method to choose the number of factors) and Varimax rotation (consider that the factors are not correlated). The use of Little Jiffy has been heavily criticized and its use is not recommended, as it may lead to the acceptance of erroneous factor models [67].

From a theoretical point of view, the time/control factor is related to behavioral symptoms (e.g., neglect household chores to spend more time online), while the stress/compensate factor is made up of cognitive and affective symptoms (e.g., block out disturbing thoughts about your life with soothing thoughts of the Internet or snap, yell, or act annoyed if someone bothers you while you are online). This bifactor model is framed within the cognitive-behavioral perspective, explaining the symptoms of Internet addiction (of varied nature) from specific and generalized uses, which simultaneously influence the symptoms [15]. The model obtained in this study theoretically differs from the models presented in the literature review, because a component (behavioral, cognitive, or affective) is not emphasized, but rather, the components are worked on simultaneously.

Convergent evidence from the IAT was also provided, finding statistically significant correlations with the hours of daily Internet use, and time/control and total Internet addiction presented a recommended minimum effect size that represents practical significance in social science data. These results were like those obtained by other researchers [38,41,45,50]. Additionally, the IAT negatively correlates with social skills measures (total score and self-expression, disagreement, and assertiveness factors). Previous studies also report these relationships with similar degrees of correlation. Internet addiction is associated with greater difficulties in social skills, probably since the emotional burden produced by being connected to the Internet interferes with social aspects. In this way, the development of social skills is left aside due to the few social interactions that the subject experiences, since most of his time is online [2,71].

Regarding the reliability of the scores on the IAT, the ordinal alpha coefficient showed acceptable levels for the general factor and the specific factors. On this point, most of previous studies coincide, including meta-analytical studies [65].

Regarding the limitations of the study, the main one focuses on the size and variety of the sample. Regarding the first aspect, although globally, the sample size is justified in an a priori statistical power analysis, the number of participants within the groups of sociodemographic variables is small, which limits the possibility of carrying out additional analyses in the items. For example, knowing the differential functioning of items or knowing the factorial invariance of the IAT. Regarding the variety of the sample, the students belonged to a public university; therefore, they share various characteristics that make it a homogeneous group, and therefore, the variability in the responses to the items was low.

To know and deepen other characteristics of the IAT, future studies should focus their objectives on analyses that provide evidence of its clinical utility. In this way, the appropriate cut-off points should be determined to be able to classify people addicted to the Internet and, in turn, evaluate the intensity of this addiction. Likewise, it is necessary to previously know how the instrument works in people with a presumption of Internet addiction, as well as to what extent it is related to other tests that measure clinical constructs (e.g., depression or anxiety), being relevant measures to obtain validity evidence based on relations to other variables. Therefore, working with clinical samples is a necessity, since the IAT could have different uses in the diagnosis and treatment of Internet addiction.

Likewise, the study of the IAT in non-academic populations must be accompanied by a review of the content of the items, since some of them may only be valid for the population of university students, for example, item 6 “How often do your grades or school work suffer because of the amount of time you spend online? ”, being, in this case, the rewriting of the item or its exclusion from the test. Additionally, given the complexity of the IAT structure, other multivariate techniques could be tried to corroborate what was found here or to propose more stable structures. Techniques, such as network analysis, exploratory structural equation modeling (ESEM), or Bayesian approaches to factor analysis, would help in this regard. Regarding reliability, it is relevant to obtain evidence on temporal stability (test-retest reliability), particularly useful in the IAT, due to the high variability that scores in this type of test can have.

## 6. Conclusions

This study represents a contribution to the study of the IAT in Latin America, where it has been little studied, unlike other contexts, such as Europe or Asia. The findings indicate that the IAT, in the sample of Peruvian university students, is made up of a general factor and two specific factors (time/control and stress/compensate), or a bifactor model. Likewise, added to the validity evidence based on the internal structure, the IAT showed evidence based on the relationship with other variables (average hours per day on the Internet). On the other hand, the reliability of the scores was acceptable.

The results lead to the conclusion that the scores in the IAT have evidence of validity and reliability for its use. This has implications for both researchers and those who are primarily involved in the patient practice. For researchers, the IAT constitutes an instrument that would allow studies on Internet addiction to be carried out, for example, knowing its prevalence in certain groups, identifying the factors associated with its genesis and evolution in people, or knowing the degree of sensitivity and specificity with which one can diagnose a person with Internet addiction. For professionals, the IAT is a tool that would help diagnose Internet addiction, and it would also allow evaluation of the effects produced by a treatment or therapy that seeks to decrease the level of Internet addiction.

## Figures and Tables

**Table 1:** Previous studies on psychometric properties of the Internet Addiction Test (IAT).

Study	Country	Sample Size	Factors	Data Analysis	Items	Reliability (α)
1. Widyanto et al. (2004) [24]	-	86	6	PCA	20	>0.50 ^c^
2. Ngai (2007) [25]	Hong Kong	988	4	PCA	20	>0.60 ^c^
3. Chang et al. (2008) [26]	Hong Kong	410	3	PCA, CFA ^a^	20	>0.80 ^d^
4. Khazaal et al. (2008) [27]	France	246	1	EFA, CFA	20	0.93
5. Widyanto et al. (2011) [28]	-	225	3	PCA	20	-
6. Panayides et al. (2012) [29]	Cyprus	604	1	PCA	20	0.89
7. Jelenchick et al. (2012) [30]	United States	215	2	EFA	20	>0.80 ^c^
8. Barke et al. (2012) [31]	Germany	841	2	PCA, CFA	20	0.89
9. Puerta-Cortés et al. (2012) [32]	Colombia	1117	3	PCA	20	0.89
10. Faraci et al. (2013) [33]	Italy	485	1, 2	EFA, CFA	20, 18	>0.70 ^c^
11. Watters et al. (2013) [34]	Canada	1948	2	CFA ^b^	20	0.93
12. Pawlikowski et al. (2013) [35]	Germany	1049	2	PCA, CFA	11	>0.80 ^c^
13. Lee et al. (2013) [36]	Korea	279	4	PCA	20	0.91
14. Hawi (2013) [37]	Lebanon	817	1	PCA, CFA	20	0.92
15. Lai et al. (2013) [38]	Hong Kong	844	3	CFA ^a^	20	0.93
16. Pontes et al. (2014) [39]	Portugal	593	1	CFA	20	0.90
17. Karim et al. (2014) [40]	Bangladesh	177	4	PCA	18	0.89
18. Tsimtsiou et al. (2014) [41]	Greece	151	3	EFA	20	0.91
19. Chong et al. (2015) [42]	Malaysia	162	5	PCA	20	0.91
20. Fernández-Villa et al. (2015) [43]	Spain	963	2	EFA, CFA	19	0.91
21. Lu et al. (2015) [44]	Malaysia	104	6	EFA	20	0.93 ^e^
22. Dhir et al. (2015) [45]	India	1914	1	EFA, CFA	20	0.88
23. Lai et al. (2015) [46]	Hong Kong, Japan and Malaysia	2535	3	CFA	20	-
24. Fioravanti et al. (2015) [47]	Italy	840	2	EFA, CFA	20	>0.80 ^c^
25. Hawi (2015) [48]	Poland	1245	2	PCA, CFA	20	0.90
26. Kaya (2016) [49]	Turkey	990	4	EFA, CFA	20	0.92
27. Servidio (2017) [50]	Italy	659	2	PCA, CFA	18	0.89
28. Boysan et al. (2017) [51]	Turkey	455	1	PCA, CFA	20	0.93
29. Samaha et al. (2018) [52]	Lebanon	256	4	EFA, CFA	19	0.91
30. Waqas et al. (2018) [53]	Pakistan	522	1	EFA, CFA	20	0.90
31. Neelapaijit et al. (2018) [54]	Thailand	324	3	EFA, CFA	20	0.89
32. Tsermentseli et al. (2018) [55]	Greece	725	3	EFA, CFA ^b^	19	>0.70 ^f^
33. Hernández et al. (2018) [56]	Chile	425	2	CFA	10	0.85
34. Černja et al. (2019) [57]	Croatia	352	3	PCA, CFA	20	0.91
35. Tudorel et al. (2019) [58]	Romania	421	2	EFA, CFA	20	0.86
36. Ndasauka et al. (2019) [59]	Pakistan	506	4	EFA	20	0.88
37. Yaffe et al. (2019) [60]	Israel	180	2	PCA, CFA	18	>0.70 ^c^
38. Talwar et al. (2019) [61]	Malaysia	307	3	PCA, CFA	19	>0.70 ^c^
39. Lu et al. (2019) [62]	Malaysia	1120	4	EFA, CFA	17	-

Note. ^a^ Hierarchical model; ^b^ Bifactor model; ^c^ α coefficients of the factors; ^d^ Construct reliability; ^e^ Rasch model (person reliability); ^f^ ω coefficients of the factors; PCA = Principal Component Analysis; EFA = Exploratory Factor Analysis; CFA = Confirmatory Factor Analysis.

**Table 2:** Sociodemographic characteristics of participants (*n* = 227).

Characteristic	*n*	%
Gender		
Male	97	42.70
Female	130	57.30
Year of study		
First	24	10.60
Second	68	30.00
Third	63	27.80
Fourth	42	18.50
Fifth	30	13.20
Academic discipline		
Health Sciences	41	18.10
Humanities	30	13.20
Social Sciences	27	11.90
Basic sciences	43	18.90
Engineering	45	19.80
Economic-Business	41	18.10

**Table 3:** Item analysis for the Internet Addiction Test (IAT).

Item	M	SD	Sk	Ku	Item-Rest Correlation	Floor (%)	Ceiling (%)
Item 1	3.084	1.200	0.115	−0.989	0.378	8	16
Item 2	2.066	0.902	0.734	0.375	0.570	28	1
Item 3	1.885	1.146	1.118	0.203	0.381	53	4
Item 4	1.670	0.826	1.329	1.882	0.381	51	1
Item 5	1.925	1.021	1.092	0.775	0.560	42	3
Item 6	1.828	0.908	1.013	0.437	0.532	43	0
Item 7	3.084	1.211	0.168	−1.016	0.371	7	17
Item 8	1.797	0.889	0.968	0.163	0.591	45	0
Item 9	1.656	0.860	1.506	2.404	0.471	53	1
Item 10	1.753	0.913	1.024	0.245	0.465	51	0
Item 11	1.855	0.898	0.798	−0.072	0.496	43	0
Item 12	1.626	0.900	1.489	1.868	0.451	59	1
Item 13	1.771	0.960	1.275	1.200	0.527	50	2
Item 14	1.877	1.006	1.053	0.442	0.515	45	2
Item 15	1.502	0.772	1.571	2.251	0.684	64	0
Item 16	2.286	1.094	0.833	0.149	0.443	24	6
Item 17	2.093	1.066	0.906	0.138	0.558	33	3
Item 18	1.736	1.000	1.415	1.409	0.538	54	2
Item 19	1.414	0.796	2.275	5.499	0.626	72	1
Item 20	1.352	0.658	2.176	5.640	0.512	73	0

Note. M = Mean; SD = Standard Deviation; Sk = Skewness; Ku = Kurtosis.

**Table 4:** Confirmatory factor analysis for the IAT.

Model	SSχ^2^	*df*	SSχ^2^/*df*	RMSEA (90% CI)	CFI	TLI	SRMR	WRMR
1. One-factor [29,45,51,53]	387.285	161	2.405	0.079 (0.069; 0.089)	0.890	0.871	0.092	1.168
2. Khazaal et al. (2008) [27]	449.357	169	2.659	0.086 (0.076; 0.095)	0.864	0.847	0.101	1.288
3. Widyanto et al. (2011) [28]	423.779	167	2.358	0.082 (0.073; 0.092)	0.876	0.859	0.095	1.244
4. Jelenchick et al. (2012) [30]	393.896	169	2.331	0.077 (0.067; 0.087)	0.891	0.878	0.092	1.189
5. Barke et al. (2012) [31]	382.215	167	2.289	0.076 (0.066; 0.086)	0.896	0.881	0.091	1.163
6. Watters et al. (2013) [34]	285.741	154	1.855	0.062 (0.050; 0.073)	0.936	0.921	0.073	0.939
7. Hawi (2013) [37]	462.205	166	2.784	0.089 (0.079; 0.099)	0.857	0.836	0.102	1.311
8. Lee et al. (2013) [36]	340.237	164	2.075	0.069 (0.059; 0.079)	0.915	0.901	0.084	1.086
9. Pontes et al. (2014) [39]	437.216	168	2.602	0.084 (0.075; 0.094)	0.870	0.853	0.099	1.267
10. Tsimtsiou et al. (2014) [41]	375.281	167	2.247	0.074 (0.064; 0.084)	0.899	0.885	0.090	1.156
11. Fioravanti et al. (2015) [47]	390.065	165	2.364	0.078 (0.068; 0.088)	0.891	0.875	0.093	1.182
12. Hawi (2015) [48]	342.871	169	2.029	0.067 (0.057; 0.078)	0.916	0.905	0.085	1.097
13. Dhir et al. (2015) [45]	437.235	166	2.634	0.085 (0.075; 0.095)	0.869	0.850	0.099	1.266
14. Waqas et al. (2018) [53]	475.709	170	2.798	0.089 (0.080; 0.099)	0.852	0.835	0.104	1.336
15. Tudorel et al. (2019) [58]	388.449	167	2.326	0.077 (0.067; 0.087)	0.893	0.878	0.092	1.176
16. Černja et al. (2019) [57]	399.818	167	2.394	0.079 (0.069; 0.088)	0.887	0.872	0.093	1.200

Note. RMSEA = Root Mean Square Error of Approximation; CI = Confidence Interval; CFI = Comparative Fit Index; TLI = Tucker–Lewis Index; SRMR = Standardized Root Mean Square Residual; WRMR = Weighted Root Mean Square Residual.

**Table 5:** Interfactorial matrix, convergent evidence, and reliability of the IAT.

Variable	Time/Control	Stress/Compensate	Internet Addiction
1. Time/Control	-		
2. Stress/Compensate	0.400 ***	-	
3. Internet Addiction	0.804 ***	0.861 ***	-
4. Self-Expression	−0.117	−0.197 **	−0.331 ***
5. Defense of rights	−0.185 **	−0.189 **	−0.350 ***
6. Disagreement	−0.055	−0.205 **	−0.311 ***
7. Assertiveness	−0.054	−0.189 **	−0.196 **
8. Making requests	0.019	−0.029	−0.118
9. Starting interactions	−0.127	0.058	−0.118
10. Social Skills	−0.137 *	−0.186 **	−0.257 ***
11. Hours on the Internet	0.397 ***	0.112	0.337 ***
12. Ordinal Alpha	0.727	0.856	0.888

Note. * *p* < 0.05; ** *p* < 0.01; *** *p* < 0.001.

## Appendix A

English and Spanish version of the Internet Addiction Test (IAT)

1. How often do you find that you stay online longer than you intended?
  ¿Con qué frecuencia se conecta a internet más de lo previsto?
2. How often do you neglect household chores to spend more time online?
  ¿Con qué frecuencia descuida las actividades de la casa para estar más tiempo conectado?
3. How often do you prefer the excitement of the internet to intimacy with your partner?
  ¿Con qué frecuencia prefiere más la emoción que le produce estar conectado a la intimidad con su pareja o la relación directa con sus amigos?
4. How often do you form new relationships with fellow online users?
  ¿Con qué frecuencia forma nuevas relaciones con usuarios de Internet?
5. How often do others in your life complain to you about the amount of time you spend online?
  ¿Con qué frecuencia las personas cercanas a usted se quejan por la cantidad de tiempo que permanece conectado?
6. How often do your grades or school work suffer because of the amount of time you spend online?
  ¿Con qué frecuencia sus calificaciones o actividades académicas se afectan negativamente por la cantidad de tiempo que permanece en Internet?
7. How often do you check your email before something else that you need to do?
  ¿Con qué frecuencia revisa su correo electrónico antes de realizar otra tarea que necesita hacer?
8. How often does your job performance or productivity suffer because of the internet?
  ¿Con qué frecuencia el tiempo que pasa en Internet afecta negativamente su desempeño o productividad en el trabajo?
9. How often do you become defensive or secretive when anyone asks you what you do online?
  ¿Con qué frecuencia está a la defensiva o se muestra reservado cuando alguien le pregunta qué hace en Internet?
10. How often do you block out disturbing thoughts about your life with soothing thoughts of the internet?
  ¿Con qué frecuencia bloquea los pensamientos desagradables de su vida con pensamientos agradables relacionados con Internet?
11. How often do you find yourself anticipating when you will go online again?
  ¿Con qué frecuencia anticipa cuando estará conectado de nuevo?
12. How often do you fear that life without the internet would be boring, empty, and joyless?
  ¿Con qué frecuencia teme que la vida sin Internet sería aburrida, vacía o triste?
13. How often do you snap, yell, or act annoyed if someone bothers you while you are online?
  ¿Con qué frecuencia se enoja si alguien lo molesta mientras está conectado?
14. How often do you lose sleep due to being online?
  ¿Con qué frecuencia se queda sin dormir por conectarse durante la noche?
15. How often do you feel preoccupied with the internet when off-line, or fantasize about being online?
  ¿Con qué frecuencia se siente preocupado por no estar conectado o imagina estarlo?
16. How often do you find yourself saying “just a few more minutes” when online?
  ¿Con qué frecuencia dice: “unos minutos más”, cuando está conectado?
17. How often do you try to cut down the amount of time you spend online and fail?
  ¿Con qué frecuencia trata de disminuir el tiempo que pasa en Internet y no lo logra?
18. How often do you try to hide how long you’ve been online?
  ¿Con qué frecuencia intenta ocultar el tiempo que permanece conectado?
19. How often do you choose to spend more time online over going out with others?
  ¿Con qué frecuencia prefiere pasar más tiempo en Internet que salir con otras personas?
20. How often do you feel depressed, moody, or nervous when you are off-line, which goes away once you are back online?
  ¿Con qué frecuencia se siente deprimido, malhumorado o nervioso cuando no está conectado, pero se siente mejor cuando se conecta de nuevo?
